# Effect of reduced plant height on drought tolerance in rice

**DOI:** 10.1007/s13205-016-0542-3

**Published:** 2016-10-12

**Authors:** Asadollah Ahmadikhah, Amir Marufinia

**Affiliations:** 1 Department of Biotechnology, Faculty of New Technologies, Shahid Beheshti University, Tehran, Iran; 2 Department of Plant Breeding and Biotechnology, Gorgan University of Agricultural Sciences and Natural Resources, Gorgan, Iran

**Keywords:** Drought, Dwarf, Morpho-physiological, Mutation, Rice, Yield loss

## Abstract

Drought stress due to water deficit is a major problem of rice cultivation as a most drought-sensitive crop plant. A rice mutant line (MT58) was developed after mutagenesis of cv. Neda by ethyl methane sulfonate (EMS) and selected for dwarfism (18 cm shorter than Neda). The extent of its molecular changes relative to parental cultivar was assessed by SSR and ISSR markers, and the response of the line along with parental cultivar and another mutant line (MTA) to mild and severe water deficit, was evaluated in a field experiment. A molecular assessment using 41 SSR markers showed that dwarf line MT58 had significant molecular difference with two other lines. ISSR assay also proved the considerable mutational effect of EMS on two mutant lines compared with the original wild line. Field experiments revealed that limited irrigation caused mild-to-severe decrease in all the studied traits, including chlorophyll contents. In mild water-stress mutant line, MT58 showed a low (3 %) yield loss as compared with cultivar Neda with a high (14 %) yield loss. Interestingly, in severe water-stress mutant line, MT58 showed a low (19 %) yield loss as compared with mutant line MTA and cv. Neda with high (33 and 31 %, respectively) yield loss. In severe stress, mutant MT58 had the highest values of panicle length, total kernels per panicle, fertile kernels, and chlorophyll contents, while cv. Neda had the highest values of plant height, tiller number, and plant yield, and reduction in chlorophyll content at drought stress condition was correlated with yield loss (0.64 and 0.697 for chl.*a* and chl.*b*, respectively). The results of this research obviously confirm that mutant line MT58 despite of its stunt figure shows a low yield loss due to drought stress and hence is a promising line for cultivation under drought condition.

**Electronic supplementary material:**

The online version of this article (doi:10.1007/s13205-016-0542-3) contains supplementary material, which is available to authorized users.

## Introduction

Abiotic stresses especially drought can affect the physiological status of an organism and have adverse effects on growth, development, and metabolism (Chutia and Borah 2012). Drought is an abiotic stress which affects plants at various levels and stages of their life period. This abiotic stress not only affects plant–water relations through the reduction of water content, turgor, and total water, but it also affects stomatal closure, limits gas exchange, reduces transpiration, and disturbs photosynthesis (Razak et al. 2013). Negative effects of water deficit on mineral nutrition and metabolism decrease the leaf area and alter assimilate partitioning among the plant organs (Zain et al. 2014).

Drought stress is of high importance, particularly for drought-sensitive plants, such as rice. Rice varieties have differential responses to abiotic stresses because of the complexity of interactions between stress factors and various molecular, biochemical, and physiological processes that affect plant growth and development (Zhu 2002; Wadhwa et al. 2010). Drought stress due to water deficit is a common constraint in upland cultivation systems of plants (Zain et al. 2014). More than the 20 million hectares of rain-fed lowland rice worldwide suffer water deficit at different growth stages (Cabangon et al. 2002; Quampah et al. 2011; Zain et al. 2014). Drought stress reduces the rice growth and severely affects different traits, such as seedling biomass, stomatal conductance, photosynthesis, starch metabolism, and plant–water relations (Sarkarung et al. 1997; Jaleel et al. 2009; Farooq et al. 2009; Quampah et al. 2011). Pantuwan et al. (2000) reported that grain yield of some rice varieties was reduced by up to 81 % under drought condition and this reduction depended on timing, duration, and severity of the plant water stress. Cha-um et al. (2010) reported differential responses of rice genotypes to water deficit. They observed when rice genotyped exposed to water deficit, panicle length and fertile grains in two tolerant varieties were not significantly decreased, leading to greater productivity than in two sensitive cultivars (Cha-um et al. 2010).

Influence of the environmental stresses on growth and yield can be estimated by measurement of photosynthetic traits, such as chlorophyll content and chlorophyll fluorescence parameters, since these traits have a close correlation with carbon exchange rate (Guo and Li 2000). Energetic status of the chloroplast increases as a consequence of the water stress which has a direct relationship to that of increased amount of total chlorophyll and Chla and Chlb among the stressed induced verities (Ranjbarfordoei et al. 2000). Rice is one of the most drought-susceptible crops, especially at the reproductive stage (Agarwal et al. 2016). It was reported that at rain-fed conditions, water deficit has a serious effect, especially at the booting stage, during which plants are particularly drought-susceptible, leading to low-crop productivity (Pantuwan et al. 2002).

Since water availability will be a major constraint for paddy rice productivity in the near future, we studied the response of a dwarf mutant line of rice (recently developed in our research group using mutagenesis by ethyl methane sulfonate, EMS) along with its parental cultivar to water deficit aiming to evaluate effect of dwarfism on morpho-physiological traits, particularly plant yield in a field experiment.

## Materials and methods

### Plant material

An elite high-yielding rice cultivar, Neda (widely cultivated at North of Iran), was mutagenized using ethyl methane sulfonate (EMS) and a mutant line with dwarfism was selected from M_2_ population (Shojaeian 2011). In addition, another mutant line with improved yield was included in the study. Original cv. Neda along with these two promising mutant lines (MT58 and MTA) was evaluated under drought stress.

### Drought induction and morpho-physiological measurements

Field experiment was conducted in research farm of Gorgan University of Agricultural Sciences and Natural Resources in 2013 in a split block design with three replications. Three genotypes [Neda, MT58 (M_5_), and MTA (M_5_)] were sown in three blocks of irrigation (S_0_: full irrigation; S_1_: 1 day irrigation followed by 1 day no irrigation; S_2_: 1 day irrigation followed by 2 days no irrigation). Plants of each genotype were transplanted in plots of five rows with 25 cm between rows and 25 cm spacing between hills. A single 30-day-old seedling was transplanted per hill. At appropriate times, some important traits were evaluated, including morphological traits [plant height (PLH), panicle length (PL), total kernels per panicle (TK), fertile kernels (FK) per panicle, tiller number (TN), 100-kernel weight (HKW), and plant yield (PY)] and physiological characters [chlorophyll *a* (Chl.*a*), chlorophyll *b* (Chl.*b*), and total chlorophyll (Chl.*a* + *b*) contents]. Chlorophyll contents were determined by taking fresh leaf samples (0.1 g) from flag leaves three times in reproductive phase (start of panicle emergence, 1 day after panicle emergence and 2 days after panicle emergence). The samples were homogenized with 5 mL of acetone (80 % v/v) using pestle and mortar and centrifuged at 5000 rpm. The absorbance was measured with a UV/visible spectrophotometer at 663.6 and 646.6 nm, and chlorophyll contents were calculated using the equations proposed by Porra (2002).

### Molecular assessments

Cultivar Neda along with two promising mutant lines (MT58 with stunt figure and MTA) was evaluated at molecular level using 41 simple sequence repeat (SSR) primer pairs (http://www.gramene.org; RM522, RM7300, RM6128, RM272, RM134, RM3510, RM5373, RM311, RM1146, RM3233, RM1, RM7180, RM7241, RM207, RM131, RM25, RM215, RM158, RM417, RM320, RM516, RM502, RM332, RM317, RM339, RM457, RM687, RM50, RM206, RM242, RM527, RM566, RM3873, RM283, RM505, RM481, RM106, RM159, RM171, RM412, and RM1108). The primer sequences, genomic locations, and other useful information are presented in Supplementary file S1. In addition, ten inter simple sequence repeat (ISSR) primers were used for genotyping of the studied lines (Table 1). Polymerase chain reaction (PCR) mixture was prepared in a 0.2-mL tubes and each single reaction included 5 µL of deionized water, 6-µL PCR Master Mix (CinaClone Co.), 0.25 µL of each primer (10 pg), and 0.75 µL of DNA template (15 ng). In the case of SSR assays, the PCR amplification started at 94 °C for 4 min and continued for 35 cycles, including denaturation at 94 °C for 35 s, annealing at 55 °C for 35 s, and extension at 72 °C for 40 s. The synthesis was completed at 72 °C for 7 min. The PCR products were separated by electrophoresis in 3.5 % agarose gel and 1ξ TBE buffer containing 0.5 µg mL^−1^ ethidium bromide. In the case of ISSR assays, the PCR amplification started at 94 °C for 4 min and continued for 35 cycles, including denaturation at 94 °C for 35 s, annealing at 47 °C for 35 s, and extension at 72 °C for 2 min. The synthesis was completed at 72 °C for 7 min. The PCR products were separated by electrophoresis in 1.5 % agarose gel and 1ξ TBE buffer containing 0.5-µg mL^−1^ ethidium bromide. Electrophoresis gels were photographed under ultraviolet light by a Gel-Doc system (BioRad, USA).

**Table 1:** ISSR primers used in the study and their polymorphic products

No.	Primer symbol	Sequence (5′-3′)	No. of polymorphic bands
1	ISSR1	(GA)7-RG	3
2	ISSR2	(CA)7-YC	4
3	ISSR3	(AG)8-T	5
4	ISSR4	(AG)8-YC	4
5	ISSR5	(GT)8-YC	1
6	ISSR6	(AC)8-YG	1
7	ISSR7	(TG)8-RC	1
8	ISSR8	(AT)7-RC	0
9	ISSR9	(CA)7-YG	3
10	ISSR10	(CA)8-RC	4

R and Y in primer sequence indicate degeneration at 3′ end. R: A or G; Y: C or T

### Data analysis

Morphological field data were analyzed using the SAS software (version 9.3) (SAS Institute 2009). Mean comparisons were performed using Duncan’s multiple range test (*P* < 0.05). SSR and ISSR score data were analyzed using NTSYSpc (version 2.2). A cluster analysis based on marker data was done in NTSYSpc (version 2.2).

## Results

### SSR and ISSR assays

**Fig. 1:**
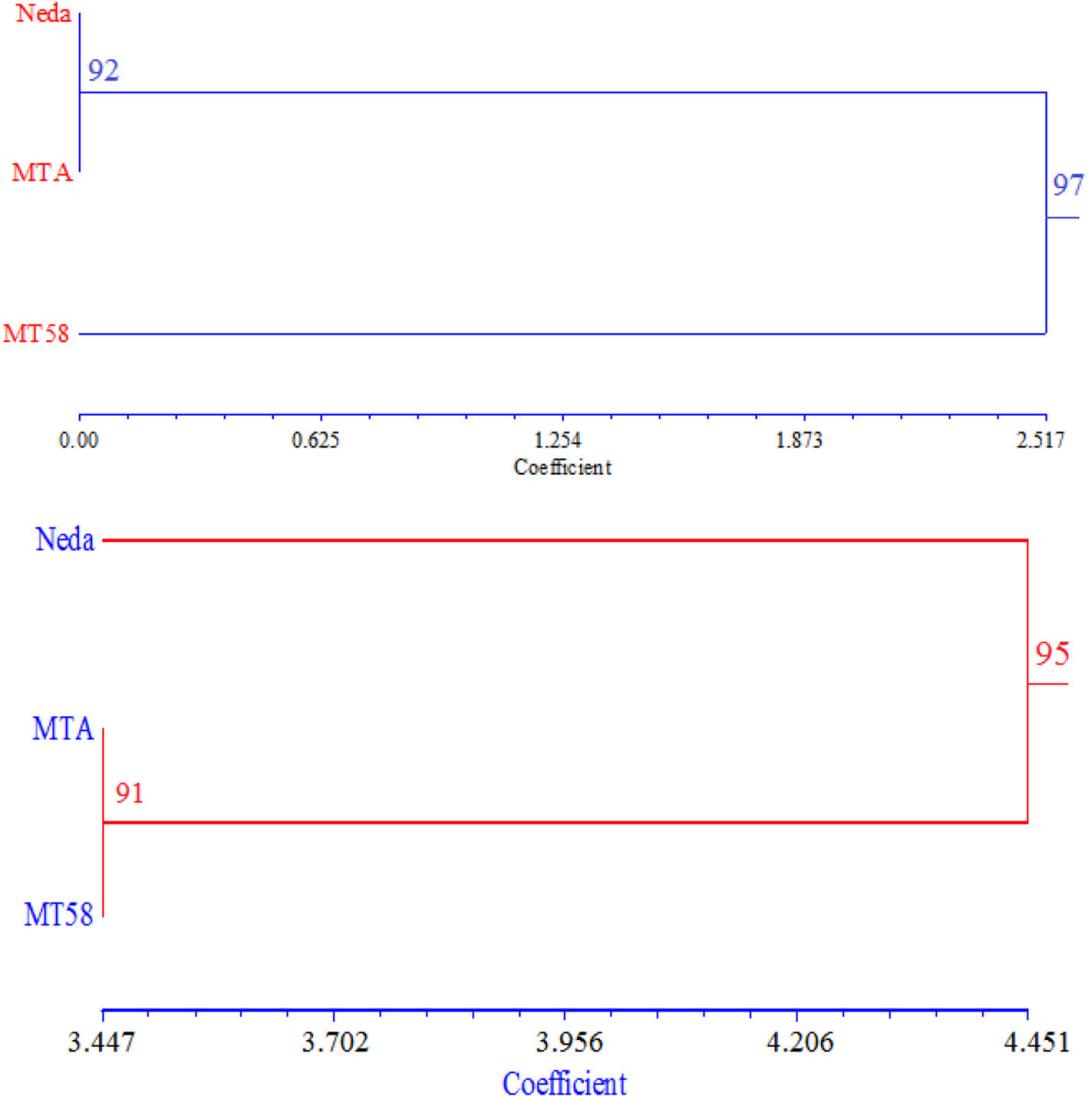
Dendrograms of the studied genotypes using the UPGMA method based on SSR (*top*) or ISSR (*bottom*) markers

At all SSR loci, wild-type line Neda, and mutant line MTA showed identical banding patterns. However, mutant line MT58 showed different banding patterns in 7 out of 41 SSR loci, including RM1, RM3510, RM3233, RM1146, RM206, RM3873, and RM505. Most of these polymorphic SSRs have dinucleotide AG or GA repeated motifs; the exceptions are RM505 and RM3510 with dinucleotide CT repeat (for more details, see supplementary file S1) Cluster analysis placed the mutant line MT58 in a separate group and two lines Neda and MTA in another group with >90 % bootstrap support (Fig. 1 top); in addition, the grouping was validated by a high cophenetic correlation coefficient (*r* = 0.968). However, based on ISSR markers, of 54 produced bands by 10 ISSR primers, 26 polymorphic bands (48.1 %) were observed between three genotypes. Cluster analysis differentiated the two mutant lines from wild-type line Neda and they were placed in a separate group with a high bootstrap support (Fig. 1 bottom); in this case, also the grouping was validated by a high cophenetic correlation coefficient (*r* = 0.962).

### Effect of water deficit on morphological traits

Analysis of variance (ANOVA) showed that water stress significantly affected morphological traits, including plant height (PLH) and plant yield (PY) (Table 2), indicating that these traits were highly affected by irrigation regime. Genotype effect was significant for all morphological traits except for tiller number and plant yield. Effect of stress × genotype interaction was significant only for plant height.

**Table 2:** Analysis of variance for morphological traits in different irrigation regimes

Source	*df*	PLH	PL	TK	FK	HKW	Till	PY
Rep	2	0.55	0.57	56.45	44.20	0.01	4.18	17.39
Stress	2	105.72**	1.65	780.81	734.04	0.02	12.65	291.28*
E_a_	6	0.17	0.49	302.10	507.07	0.01	5.37	53.92
Genotype	2	847.12**	5.22**	3310.90**	2210.47**	0.35**	2.11	14.62
Stress × genotype	4	5.42**	0.12	221.31	61.58	0.01	2.97	13.82
E_b_	9	0.43	0.26	119.90	164.96	0.01	1.22	14.91

* and ** indicating significant differences at 5 and 1 % level of probability, respectively

Mean’s comparisons revealed that irrigation regimes had significant differences in all the studied traits except for 100-kernel weight (Table 3). As expected, maximum performance of all traits was observed in normal irrigation (S_0_). Mild water stress (S_1_) and severe water stress (S_2_), respectively, had moderate and severe negative effects on all the studied traits. Particularly, severe water deficit (S_2_) compared with normal irrigation significantly decreased plant height (8 cm), total kernels per panicle (18 kernels), tiller number (2 tillers), and plant yield (12 g/plant).

**Table 3:** Mean’s comparisons of different traits as influenced by water stress and genotype

	PLH	PL	TK	FK	HKW	Till	PY
Water stress							
S_0_	100.67^a^	24.34^a^	134.58^a^	119.20^a^	2.59^a^	16.69^a^	43.91^a^
S_1_	96.56^b^	23.71^b^	125.96^ab^	109.60^ab^	2.54^a^	16.82^a^	39.45^b^
S_2_	92.89^c^	23.49^b^	116.39^b^	100.83^b^	2.52^a^	14.51^b^	31.97^c^
Genotype							
Neda	102.78^a^	23.33^b^	113.82^b^	102.49^b^	2.67^a^	16.31^a^	39.81^a^
MTA	102.19^a^	23.47^b^	115.24^b^	99.4^b^	2.66^a^	15.57^a^	37.94^a^
MT58	85.00^b^	24.73^a^	147.86^a^	127.74^a^	2.31^b^	16.14^a^	37.59^a^

Values with common letters have not significant differences at 5 % level of probability

Mean’s comparisons for different genotypes showed that the three studied genotypes showed identical performance for tiller number and plant yield. Elite cultivar Neda along with mutant line MTA had higher performance for plant height and 100-kernel weight, while mutant line MT58 had higher performance for panicle length, total kernels per panicle, and fertile kernels, and showed significant dwarfism (~18 cm) relative to two other genotypes (Table 3).

**Fig. 2:**
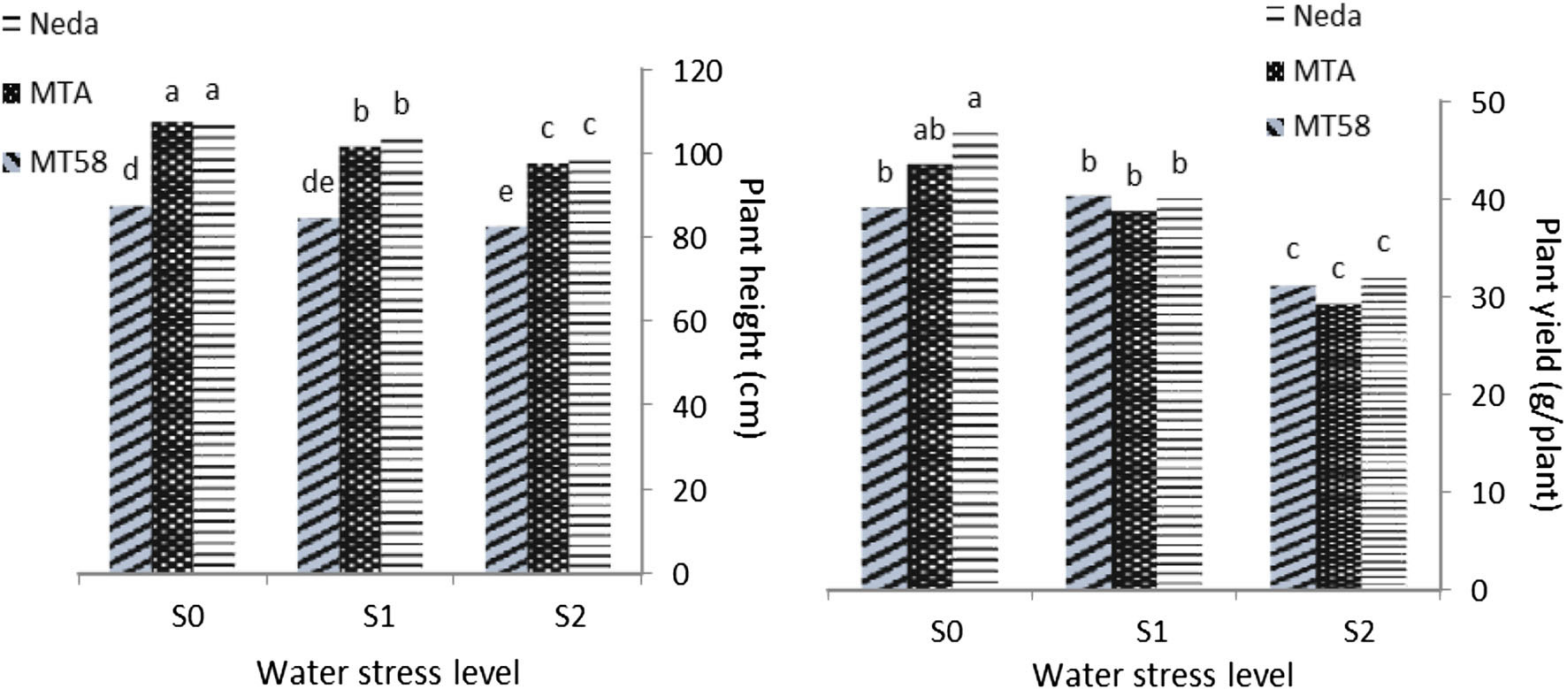
Response of studied genotypes to water deficit. *Left* plant height, *right* plant yield, *S*_*0*_ normal irrigation, *S*_*1*_ 1 day irrigation followed by 1 day no irrigation, *S*_*2*_ 1 day irrigation followed by 2 days no irrigation. Columns with *common letters* have not significant differences at 5 % level of probability

Further analysis on the stress × genotype interactions showed that in normal irrigation (S_0_), cultivar Neda had highest plant height, tiller number, and plant yield (Fig. 2; Table 4), while mutant line MTA had highest 100-kernel weight. Stunt mutant line MT58 in normal irrigation had highest panicle length, total kernels per panicle, and fertile kernels, and had shortest plant height. In severe water deficit (S_2_), all genotypes showed similar trend to what observed in mild water stress in most studied traits.

**Table 4:** Mean’s comparisons of different traits as influenced by stress × genotype interaction

	PL			TK			FK			HKW			Till		
	S_0_	S_1_	S_2_	S_0_	S_1_	S_2_	S_0_	S_1_	S_2_	S_0_	S_1_	S_2_	S_0_	S_1_	S_2_
Neda	23.64^cd^	22.94^d^	23.03^cd^	119.10^cd^	111.16^d^	107.02^d^	110.58^c^	101.91^cd^	90.67^d^	2.69^ab^	2.68^ab^	2.65^ab^	17.76^a^	17.19^ab^	13.81^e^
MTA	24.03^bc^	23.62^c^	22.81^d^	118.30^cd^	130.28^c^	104.06^d^	103.88^c^	107.56^cd^	91.78^d^	2.75^a^	2.64^ab^	2.58^b^	15.78^cd^	16.1^bc^	13.55^e^
MT58	25.25^a^	24.78^ab^	24.2^ab^	167.30^a^	149.55^b^	133.63^bc^	142.28^a^	130.76^ab^	115.21^bc^	2.35^c^	2.31^c^	2.26^c^	14.85^d^	17.04^ab^	15.28^cd^

Values with common letters have not significant differences at 5 % level of probability

**Fig. 3:**
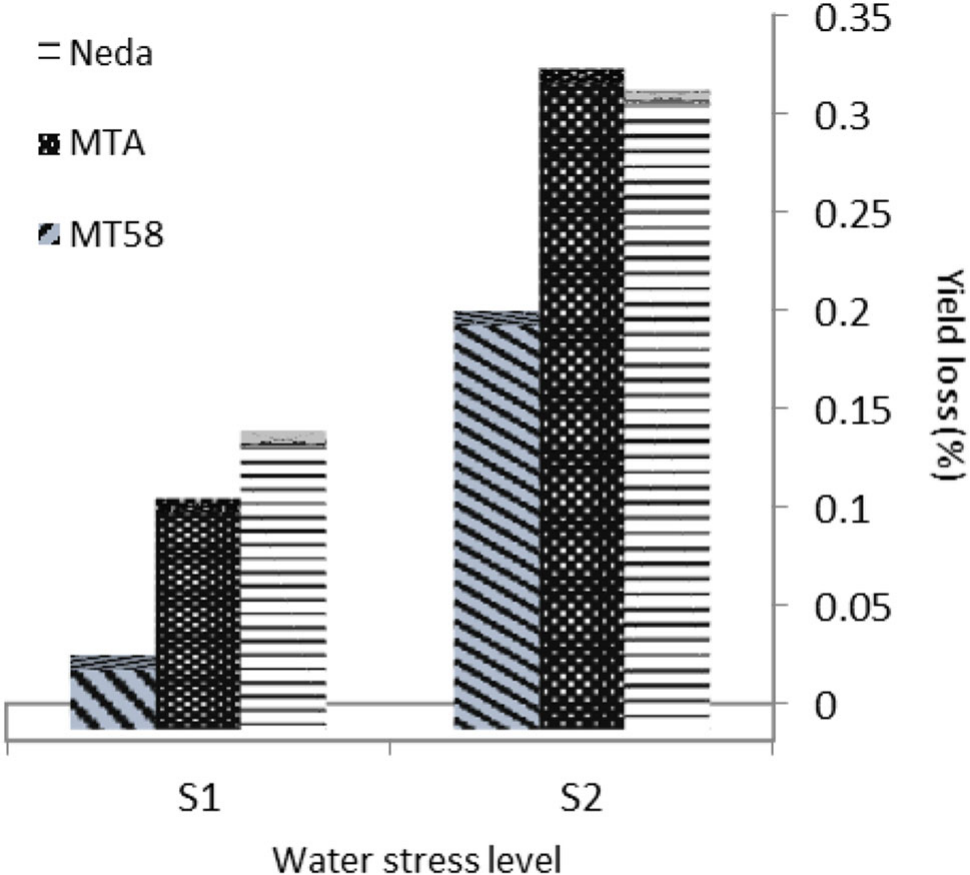
Yield loss due to water deficit of studied genotypes at two water deficit levels relative to normal irrigation condition. *S*_*1*_ 1 day irrigation followed by 1 day no irrigation, *S*_*2*_ 1 day irrigation followed by 2 days no irrigation

Assessment of yield loss due to water deficit revealed that in mild water stress (S_1_), cultivar Neda and mutant line MT58 showed highest (14 %) and lowest (3 %) yield loss, respectively (Fig. 3), while in severe water-deficit mutant lines, MTA and MT58 showed highest (33 %) and lowest (19 %) yield loss, respectively. Neda also showed relatively high yield loss (31 %).

### Effect of water deficit on chlorophyll contents

In the case of chlorophyll contents, ANOVA showed that water stress significantly affected Chl.*a* and Chl.*b* at all panicle emergence stages T1–T3 (Table 5). Genotype effect was also significant for these physiological traits, while the effect of stress × genotype interaction was significant for Chl.*a* at all panicle emergence stages and for chl.*b* at emergence stage T1 (Table 5).

**Table 5:** Analysis of variance for chlorophyll contents of flag leaf in reproductive phase in different irrigation regimes

Source	*df*	Chl.*a*			Chl.*b*		
		T1	T2	T3	T1	T2	T3
Rep	2	0.042	0.04	0.002	0.007	0.033	0.062
Stress	2	4.972**	2.818**	2.481**	4.473**	2.72**	2.514**
E_a_	6	0.034	0.010	0.019	0.014	0.013	0.063
Genotype	2	0.544**	0.321**	0.364**	0.117**	0.072*	0.243*
Stress × genotype	4	0.151*	0.132**	0.146*	0.046**	0.041	0.054
E_b_	9	0.024	0.006	0.011	0.007	0.014	0.054

T1 to T3 indicate panicle emergence, 1 day after panicle emergence, and 2 days after panicle emergence, respectively

* and ** indicating significant differences at 5 and 1 % level of probability, respectively

**Fig. 4:**
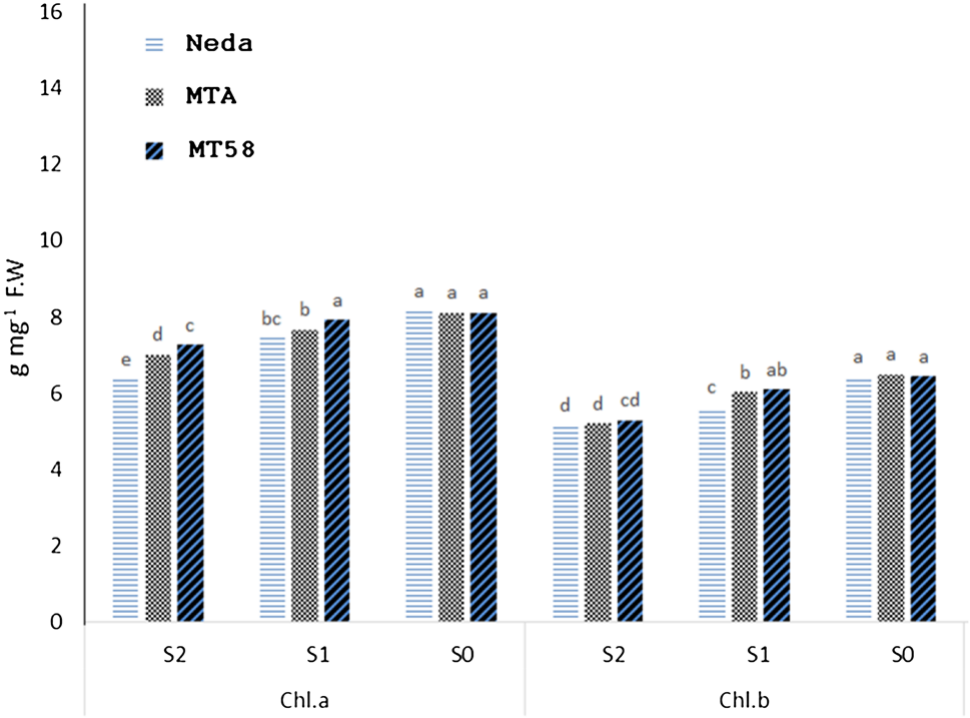
Reaction of the studied genotypes to water deficit in chlorophyll contents at T2 stage of panicle emergence

Water deficit considerably affected chlorophyll contents. Chl.*a* and Chl.*b* contents were reduced under both mild and severe drought stress (Table 6). Dwarf mutant line MT58 had higher chl.*a* contents than that of two other genotypes at all panicle emergence stages. The line also had higher chl.*b* contents at panicle emergence stages T1 and T2, but had not any difference from mutant line MTA at T3 stage. The analysis of stress by genotype interaction showed that at normal irrigation condition (S_0_), all genotypes did not differ in chlorophyll contents (as representative, Fig. 4 relates to T1 stage of panicle emergence), while at mild stress condition (S_1_), two mutant lines accumulated more Chl.*b* relative to elite cultivar Neda, and mutant line MT58 had higher Chl.*a* than two other genotypes. At severe stress condition (S_2_), mutant line MT58 accumulated more chlorophylls, although its Chl.*a* was significantly higher than that of two other genotypes.

**Table 6:** Mean’s comparisons of chlorophyll contents of flag leaf as influenced by water stress and genotype in reproductive phase

	Chl.*a*			Chl.*b*		
	T1	T2	T3	T1	T2	T3
Water stress						
S_0_	8.51^a^	8.13^a^	7.99^a^	6.81^a^	6.46^a^	6.32^a^
S_1_	8.05^b^	7.68^b^	7.54^b^	6.34^b^	5.92^b^	5.62^b^
S_2_	6.88^c^	6.88^c^	6.81^c^	5.26^c^	5.22^c^	5.14^c^
Genotype						
Neda	7.51^c^	7.32^c^	7.20^c^	5.98^c^	5.76^b^	5.51^b^
MTA	7.84^b^	7.60^b^	7.47^b^	6.20^a^	5.89^a^	5.79^a^
MT58	8.09^a^	7.77^a^	7.67^a^	6.24^a^	5.93^a^	5.75^a^

Values with common letters on each column have not significant differences at 5 % level of probability

Correlation analysis showed that among morphological traits, panicle length (PL) had highest (0.862) significant correlation with plant yield, followed by fertile kernels (FK) per panicle (0.455), and tiller number (TN) (0.45), and all chlorophyll contents had a high significant positive correlation (>0.64) with plant yield (Table 7).

**Table 7:** Relationship between the studied traits as obtained by Pearson’s correlation analysis

	PLH	PL	TK	FK	HKW	TN	PY	Chl.*a*	Chl.*b*
PLH	1								
PL	−0.514**	1							
TK	−0.599**	0.870**	1						
FK	−0.501**	0.832**	0.949**	1					
HKW	0.923**	−0.519**	−0.602**	−0.473*	1				
TN	0.145	0.412*	0.345	0.421*	0.110	1			
PY	0.352	0.450*	0.345	0.455*	0.327	0.862**	1		
Chl.*a*	0.085	0.488**	0.450*	0.461*	−0.115	0.508**	0.640**	1	
Chl.*b*	0.252	0.457*	0.410*	0.411*	0.050	0.460*	0.697**	0.913**	1

* and ** indicating significant differences at 5 and 1 % level of probability, respectively

## Discussion

In this research, we analyzed the molecular differences of an original line (Neda) and two mutant lines developed after mutagenesis of Neda line using EMS chemical mutagen. As shown, the mutation did not affect most studied SSR loci, so only 7 out of 41 loci (17.1 %) governed mutational changes in one of mutant lines (MT58). Wu et al. (2005) found no mutational changes between original rice line IR64 and nearly all morphological mutants using 12 SSR markers. In contrast, Poli et al. (2013) reported polymorphism in 6 SSR loci between IR64 and its isogenic-mutant line Nagina22. These results indicate that EMS could induce an adequate number of mutated loci in mutant MT58. In another study on wheat, Zhang et al. (2015) reported that near 21 % of SSR markers showed polymorphism between original line and an EMS-induced mutant.

In our research, in another hand, ISSR assay showed that 26 out of 54 loci (48.1 %) governed mutational changes between three studied genotypes. Wannajindaporn et al. (2014) in a research on *Dendrobium* reported a 22.5 % polymorphism in ISSR loci between original clone (Earsakul) and 28 of its mutant clones. In a work by Ahmadikhah et al. (2014) to study the EMS-induced salt tolerance in rice, 50 % of ISSR markers showed polymorphism between wild cultivar and nine mutant lines.

In water-stress assays, we found that water deficit as expected negatively imposed all evaluated morpho-physiological characters of rice plant, but the severity of loosed performance differed depending on stress level and genotype. In both mild and severe water-stresses mutant dwarf line MT58 showed lowest (3 and 19 %, respectively) yield loss. Grain yield under stress environment is the primary trait for the improvement of drought tolerance. Drought effect on seed yield is due to the relation with duration of watering from flowering until physiological maturity (Midaoui et al. 2003).

Photosynthesis is an essential process to maintain crop growth and development, and it is well known that photosynthetic systems in higher plants are most sensitive to drought stress (Falk et al. 1996). Chlorophyll is one of the major chloroplast components for photosynthesis, and relative chlorophyll content has a positive relationship with photosynthetic rate (Guo and Li 2000). Dalal and Tripathy (2012) showed that chlorophyll content was reduced under PEG-induced drought stress in rice seedlings. Ganji Arjenaki et al. (2012) also observed a reduction in chlorophyll content due to water stress at anthesis stage in wheat. Exposure to drought stress leads to a significant effect in Chlorophyll.*a* and Chlorophyll.*b* contents (Ranjbarfordoei et al. 2000). In our study, also chlorophyll contents showed considerable sensitivity to water deficit, so that with a mild water stress, all chlorophyll contents were reduced compared with normal water condition, and in a more severe water stress, their reduction was more visible (Table 3). Genotype reaction to water deficit was well observable in their chlorophyll accumulation at drought stress condition; this issue can be particularly observed for chl.*a* content (Table 3). Cha-um et al. (2010) and Mafakheri et al. (2010) reported a marked decrease in all physiological parameters due to drought stress in drought-sensitive rice and chickpea genotypes, respectively. A reason for decrease in chlorophyll content as affected by water deficit is that drought stress by producing reactive oxygen species (ROS), such as O_2_^−^ and H_2_O_2_, can lead to lipid peroxidation and, consequently, chlorophyll destruction (Mirnoff 1993; Foyer et al. 1994; Hirt and Shinozaki 2004). In addition, with decreasing chlorophyll content due to the changing green color of the leaf into yellow, the reflectance of the incident radiation is increased (Schlemmer et al. 2005).

Correlation analysis showed a clear positive relationship (>0.64) between plant yield and chlorophyll contents (Table 4), probably due to maintaining stay-green state despite of water deficit, especially in two less sensitive mutant lines, MTA and MT58. Although there is an argument about whether a higher chlorophyll content (i.e., stay-green trait) contributes to yield under drought conditions or not (Blum 1998), many studies indicated that stay-green is associated with improved yield and transpiration efficiency under water-limited conditions in sorghum, maize, wheat, barley, and rice (Benbella and Paulsen 1998; Borrell et al. 2000; Haussmann et al. 2002; Verma et al. 2004; Li et al. 2006; Cha-um et al. 2010).

## Conclusion

Based on molecular studies in this research, it can be concluded that EMS did not alter most studied SSR loci, while it introduced adequate mutational alterations in ISSR regions. Field experiments revealed that water deficit negatively affects all evaluated morpho-physiological characters of rice plant and that maintaining yield under water stress mainly is possible via continuing photosynthesis activity and staying green the leaves. With regard to all aspects of findings of our research, mutant line MT58 despite of its stunt figure did not show yield difference from its parental cultivar under drought stress.

## Electronic supplementary material

Below is the link to the electronic supplementary material.

Supplementary material 1 (XLSX 14 kb)

## Abbreviations

ANOVA: Analysis of variance
EMS: Ethyl methane sulfonate
FK: Fertile kernels
HKW: 100-Kernel weight
ISSR: Inter simple sequence repeat
PCR: Polymerase chain reaction
PL: Panicle length
PLH: Plant height
PY: Plant yield
SSR: Simple sequence repeat
TK: Total kernels per panicle
TN: Tiller number
